# Dietary Zinc Restriction and Chronic Restraint Stress Affect Mice Physiology, Immune Organ Morphology, and Liver Function

**DOI:** 10.3390/nu16223934

**Published:** 2024-11-18

**Authors:** Dorota Bederska-Łojewska, Kinga Szczepanik, Justyna Turek, Agata Machaczka, Łukasz Gąsior, Bartłomiej Pochwat, Joanna Piotrowska, Bartłomiej Rospond, Bernadeta Szewczyk

**Affiliations:** 1 Department of Neurobiology, Maj Institute of Pharmacology, Polish Academy of Sciences, ul. Smętna 12, 31-343 Kraków, Polandszewczyk@if-pan.krakow.pl (B.S.); 2 Department of Animal Nutrition and Feed Science, National Research Institute of Animal Production, ul. Krakowska 1, 32-083 Balice, Poland; kinga.szczepanik@iz.edu.pl; 3 Department of Inorganic and Analytical Chemistry, Faculty of Pharmacy, Jagiellonian University Medical College, ul. Medyczna 9, 30-688 Kraków, Poland

**Keywords:** thymus, intestines, metallothionein, oxidative stress

## Abstract

Background: Preclinical and clinical studies suggest that zinc deficiency and chronic stress contribute to depressive symptoms. Our study explores the intricate relationship between these factors by examining their physiological and biochemical effects across various organs in C57Bl/6J mice. Methods: The mice were divided into four groups: control, chronic restraint stress for 3 weeks, a zinc-restricted diet (<3 mg/kg) for 4 weeks, and a combination of stress and zinc restriction. Mice spleen and thymus weights were measured, and hematoxylin–eosin staining was conducted for liver and intestinal morphometry. Moreover, metallothionein (MT-1, MT-2, and MT-3), zinc transporter (ZnT-1), oxidative stress markers (TBARS, SOD, and GSH-Px), and zinc, iron, and copper concentrations in the liver were evaluated. Immunohistochemical analysis of the jejunum for ZIP1 and ZIP4 was also performed. Conclusions: Our findings reveal that dietary zinc restriction and chronic stress induce structural changes in the intestines and immune organs and impact metallothionein expression, oxidative stress, and liver iron and copper homeostasis.

## 1. Introduction

The importance of zinc for human health was first identified in 1963. Hematologist A.S. Prasad observed severe zinc deficiency in a 20-year-old man with a diet low in animal protein and high in phytate-rich cereals. This man exhibited anemia, growth retardation, rough and dry skin, hepatosplenomegaly, hypogonadism, and mental lethargy, which was found to be linked to insufficient zinc intake [1]. This discovery sparked further research into the role of zinc deficiency in physiological and psychological health, including depression.

In 1983, Hansen first described that depressed patients tended to have lower serum zinc levels compared to healthy individuals [2]. This finding raised the question of whether zinc deficiency in depression is a primary cause or a secondary effect. Some studies suggest that the decrease in the level of zinc can be the result of the appearance of inflammatory processes during depression, involving higher levels of prostaglandins, positive acute-phase proteins (CRP), pro-inflammatory cytokines (IL-1, IL-6, and TNF-α), and lower levels of negative acute phase proteins (albumins—zinc main transporter, and transferrin) [3].

Supporting the hypothesis of the role of zinc deficiency in the etiology of depression, preclinical studies have shown that rats and mice subjected to a chronic zinc-deficiency diet exhibited depression-like behaviors in various behavioral tests [4,5,6,7,8,9]. Animals on a low-zinc diet displayed signs of anhedonia (evaluated via the sucrose preference test (SPT) or sucrose intake test (SIT)), prolonged immobility times (as measured by the forced swimming test (FST) or tail suspension test (TST)), and altered social behaviors [4,5,6,7,9,10,11,12,13]. Depressive-like behavior observed in animals on a low-zinc diet is similar to those seen in chronically stressed animals. Stress models are often used as an important tool in studying the etiology of depression and the mechanism of action of antidepressant drugs [14]. However, the biochemical effects caused by chronic stress and a zinc-deficient diet (ZnD) in rodents are not entirely the same [13,15] Moreover, it is important to highlight that there is limited understanding of the behavioral and biological outcomes of the combination of chronic stress and ZnD in rodents. Therefore, our recent studies have aimed to determine the extent to which the behavioral or antidepressant response effects in mice exposed to ZnD and stress simultaneously differ from those observed when these factors are applied independently. Our recent paper [15] showed that ZnD with chronic stress (chronic restraint stress, CRS) induced anhedonia in mice, as evidenced by reduced sucrose preference. Interestingly, antidepressants such as imipramine and ketamine, which were effective in CRS animals, did not reverse behavioral disturbances in ZnD+CRS (ZnDCRS) animals, underscoring the need to further investigate the distinct biological changes induced by combined ZnD and CRS. These results also suggest that zinc deficiency may be a significant factor in the occurrence of drug resistance.

Beyond the nervous system, zinc deficiency and stress likely impact other organs critical to immune and metabolic regulation. In particular, we focus on the thymus, spleen, and liver to better understand systemic zinc’s role and how its deficiency, along with CRS, could disrupt immune function and zinc homeostasis.

The thymus is the organ especially susceptible to glucocorticoids. These steroid hormones produced by the adrenal glands contribute to its atrophy [16,17]. Lymphoid organs closely interact with the nervous system in a precisely coordinated way to maintain healthy physiology. The stress-induced thymic involution is reversible and stops immediately after the stress factor (physiological stress, infection, malnutrition, and cancer) is extinguished. The loss of cortical thymocytes and the reduced number of naive T cells are later followed by thymus recovery. Little is known about the mechanisms that drive thymus atrophy [18]. Except for steroid involvement, some studies reported that the TNF-α signal pathway might also be one of the possible mechanisms leading to thymocyte apoptosis.

Similarly, stress and ZnD may cause structural changes in the intestines, the largest immunological organ in the body (gut-associated lymphoid tissue-GALT) that plays a crucial role in maintaining immune homeostasis. This complex system relies on communication between epithelial cells, immune cells, and the microbiome, which results in precise immune responses to antigens [19]. In the present paper, we focused on analyzing the impact of a zinc-restricted diet and CRS on the morphometric indices of the small and large intestines. Chronically stressed rats demonstrated mucosal barrier dysfunction, damage of enterocytes, a decreased number of goblet cells, and bacteria internalization into the epithelium [20]. Luo et al., 2021 [21], showed that mice subjected to restraint stress demonstrated histological changes like loss of crypt architecture, degeneration and shortening of villi, and severe villous atrophy compared to the controls. Animals subjected to restraint stress exhibited a decrease in villus height in the duodenum and jejunum and greater crypt depth in the duodenum, jejunum, and ileum. Moreover, stress lowers the villi-to-crypt ratio in the duodenum, jejunum, and ileum. Reduced zinc content in the diet also leads to morphological changes within the intestinal epithelium [22].

Zinc homeostasis is mainly regulated by its intestinal absorption on the apical and basolateral membrane of enterocytes by zinc transporters belonging to the ZIP (SLC30) and ZnT (SLC39) family. ZIP and ZnT proteins are crucial for maintaining cellular zinc homeostasis, which is essential for numerous biochemical processes. Dysregulation of these proteins can cause various disorders associated with zinc imbalance, e.g., neurological disorders and immune dysfunction [22]. ZIP4 (Zrt/Irtb-like protein 4) is a zinc transporter protein responsible for zinc uptake into cells. ZIP4 is mainly expressed in the small intestine on the apical side of the enterocytes during zinc deficiency. During zinc deficiency, its cell expression is upregulated as a compensatory mechanism to maintain body homeostasis [23]. Chronic stress can affect various physiological processes. However, its impact on ZIP expression has not been extensively studied. ZIP1 is a transmembrane protein that transports Zn into the cytosol. Gene-deficient mouse studies of ZIP1 showed their sensitivity to dietary zinc deficiency but only during pregnancy [24]. Specific alterations to the ZIP1 transporter during zinc deficiency remain unclear. Despite the high demand for zinc in cells, free or labile zinc must be kept at very low levels [25]. Maintaining zinc cellular homeostasis requires metallothioneins (MTs). These proteins play a crucial role in regulating the storage and release of zinc ions within cells. MT is also considered a significant component of the cellular zinc buffer, which protects from sudden fluctuations in zinc ion levels that could disrupt cellular function [26]. MTs are found in different organs. One of the most critical factors in the regulation of zinc homeostasis is the liver. Zinc levels stay constant in some tissues, even during zinc deficiency. The liver represents a fast-exchangeable Zn pool, and it is susceptible to changes in serum zinc levels. The intracellular zinc metabolism is regulated through zinc transporters and metallothioneins. These two protein families closely cooperate. However, knowledge on this subject is still minimal [27]. Therefore, we decided to analyze the zinc transporter ZnT1’s concentration in the liver to determine whether it is sensitive to disturbances in zinc homeostasis. Zinc deficiency and chronic restrain stress may manifest in liver functioning and histological changes. The liver is critically important for proper brain function and during pharmacological treatment of depression. The liver–brain axis is based on several fundamental mechanisms, including the permeability of the blood–brain barrier, the functioning of the vagus nerve, epigenetic regulation, the influence of toxic metabolites, the metabolism of β-amyloid, the body’s immune response, and drug metabolism (especially with drugs requiring CYP450 enzymes) [28,29,30]. Liver dysfunction can alter the effectiveness and side-effect profile of antidepressant medications, impacting their efficacy in treating depression. Communication between the liver and the brain is inherently bidirectional, and the brain is also strongly engaged in the central neuroendocrine and neuroimmune regulation of cytochrome P450 in the liver [31]. Moreover, the liver is involved in the metabolism of GC, and its dysfunction can result in abnormal levels of these hormones, contributing to HPA axis dysregulation, which is commonly observed in depression [32,33].

In summary, these studies aimed to elucidate how the combined ZnD and CRS influence the thymus, spleen, intestines, and liver. Moreover, zinc transporters and other proteins crucial for maintaining zinc balance in cells, especially in organs like the intestines and liver, were analyzed. Investigating these systems holistically may reveal how ZnD and CRS interact to affect depressive behavior and antidepressant efficacy, potentially offering insights into treatment-resistant depression.

## 2. Materials and Methods

### 2.1. Animals and Treatments

All experiments were carried out after obtaining permission from the Local Ethical Commission for Animal Experiments at Maj Institute of Pharmacology, Polish Academy of Sciences in Krakow, which approved the experiments (permit number 87/2021).

Six-week-old male mice (C57Bl/6) were purchased from the Charles River (Germany). Animals were group-housed under controlled environmental conditions: a 12 h light/12 h dark cycle, room temperature of 19–21 °C, humidity—55 ± 10%, and free access to food and water. Cages were cleaned every week with bedding changes. Mice were weighed twice a week. Animals were adapted to diets and handled every day for a week before the beginning of the experiment. After the habituation phase, animals were randomly assigned to four different treatments. Each group consists of eight animals:

- ZnA—control group fed with pellets containing 50 mg/kg of zinc (*n* = 8);
- ZnACRS—exposed to 3 weeks of chronic restrain stress (3 h/d) and fed with pellets with 50 mg/kg of zinc (*n* = 8);
- ZnDCRS—exposed to the same stress procedure as ZnACRS and a diet containing < 3 mg/kg Zn for 4 weeks (*n* = 8);
- ZnD—received a diet with zinc level < 3 mg/kg for 4 weeks (*n* = 8).

**Figure 1:**
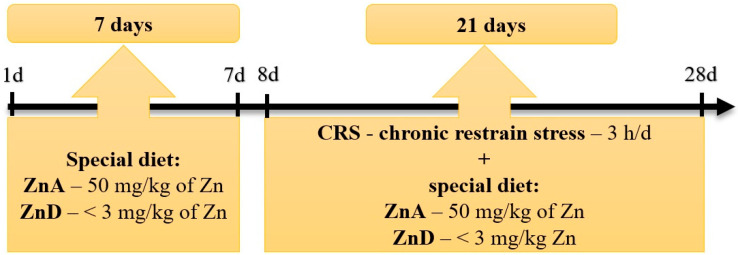
Experimental scheme of mice treatment.

Diets were purchased from Altromin GmbH, Lage, Germany. The experimental scheme of mice treatment is shown in Figure 1.

### 2.2. Chronic Restraint Stress (CRS) Protocol

The stress was performed by immobilizing each animal for 3 h daily, starting between 8:30 and 10:00 a.m. The mice were restrained for 21 days.

All animals were sacrificed using cervical dislocation. At the end of the experiment, the spleen, thymus, intestines, and liver were collected immediately after the sacrifice.

### 2.3. Histologic Analysis of Spleen, Thymus, and Intestines (n= 8)

Before histological analysis, the thymus and spleen were isolated, weighed, cleaned of surrounding tissues, and gently dried with a paper towel. Tissues from the spleen, thymus, and intestines (duodenum, jejunum, ileum, and colon) were fixed in 4% buffered formaldehyde (pH 7.0) for 24 h, dehydrated in a graded series of ethanol, cleared with a nonpolar solvent, and then embedded in paraffin (Histoplast; Thermo Shandon Limited, Cheshire, UK). Then, 4 μm thick cross-sections were cut with a microtome (Thermo Scientific Microm HM 340 E, Walldorf, Germany) and placed on one microscopic slide (Pathosolutions, Elektromed, Ankara, Turkey). Slides were stained with hematoxylin (Harris hematoxylin acidified, Elektromed) and eosin (Eosin Y alcoholic). An additional Alcian blue (Alcian blue solution, pH 2.5, Sigma Aldrich, St. Louis, MO, USA) staining was used to visualize and allow counting of the goblet cells in the intestinal sections. Stained slides were observed in brightfield using a light microscope (Axio Lab A1 430037-9010-000) with an Axiocam color 105 camera (all from Carl Zeiss, Tokyo, Japan). Collected microscopic images were examined using the following graphical analysis software: ZEN 2.3 blue edition software 3.7.97.01000 (Carl Zeiss) and ImageJ (version 1.53; US National Institutes of Health, Bethesda, MD, USA; available at http://rsb.info.nih.gov/ij/index.html; accessed 20 May 2023). For intestinal samples, the following morphometric parameters were analyzed: mucosal thickness and villi length and thickness, depth and width of crypts, and the number of goblet cells in crypts at 50 μm depth (in the large intestine) and in villi at 100 μm height (in the duodenum, jejunum, and ileum). Measurements of each variable were taken in at least several different areas of each section. The measurements were then averaged and expressed as the mean value of the calculated parameters for each mouse.

### 2.4. The Percentage of Liver Collagen Fibers (n = 8)

A sample of liver tissue (the whole right lobe of the liver) was removed from buffered formalin and then placed in histology cassettes. These cassettes were subsequently placed in a tissue processor and dehydrated through increasing alcohol concentrations (30%, 50%, 70%, 80%, 96%, and 100%). They were then permeabilized with xylene and embedded in paraffin. Slides were produced from blocks by slicing the blocks with a microtome (Microm HM 340 E, Thermo Scientific, Walldorf, Germany) into 4 μm sections. The liver preparations were stained using the Masson-Goldner trichrome technique with aniline blue to differentiate structures. Stained slides were observed in brightfield using a light microscope (Axio Lab.A1 Carl Zeiss, Oberkochen, Germany) equipped with an Olympus EP50 (Olympus, Tokyo, Japan). Collected microscopic images were examined using the following graphical analysis software: EPview software (EPview for Windows OS (64 bit) V1.4; Olympus, Tokyo, Japan) and ImageJ (version 1.53; US National Institutes of Health, Bethesda, MD, USA; available at http://rsb.info.nih.gov/ij/index.html; accessed 20 May 2023). ImageJ software version 1.53 (U.S. National Institutes of Health, Bethesda, MD, USA) was used to calculate the percentage of collagen fibers (colored blue: collagen fibers), with a random selection of regions of interest (ROIs) for each individual (4 ROIs). Quantitative assessment of liver fibrosis, specifically peritumoral fibrosis, depended on distinguishing colors between blue (collagen fibers) and red (parenchyma). The percentage of collagen fibers in the image was assessed using the Threshold function (after setting an 8-bit color scale). The percentage of collagen fibers was counted per area of ROI (percent fiber content per area of interest). Results were reported as averages for the individual.

### 2.5. Immunohistochemical Staining of the Jejunum (n = 8)

A representative jejunum sample was prepared for ZIP1 and ZIP4 analysis. Tissues were placed on adherent slides (super frost). Immunohistochemical staining with ZIP1 and ZIP4 was performed after deparaffinization in xylene and rehydration with reduced ethanol and distilled water concentrations. Heat-induced epitope recovery was performed in sodium citrate buffer (10 mM sodium citrate, pH 6.0) using a laboratory microwave oven. Sections were cooled to room temperature and washed five times in TBS buffer. Slides were incubated in an antibody-blocking solution (UltraCruz^®^ Blocking Reagent, Santa Cruz Biotechnology, Inc., Dallas, TX, USA) for 20 min at room temperature. The slides were washed five times in TBS buffer. Next, the slides were incubated for 15 min with a 10% serum solution (Normal Goat Serum, ab7481, Abcam, Cambridge, UK). The slides were then incubated with primary antibodies ZIP1 (GTX85135, GeneTex, Irvine, CA, USA, dilution 1:400) and ZIP4 (ab230099, Abcam, dilution 1:150) for 45 min at room temperature in a humid chamber. The slides were washed five times in TBS (Tris-buffered saline) buffer. The preparations were then incubated at room temperature for 40 min with Goat Anti-Rabbit IgG H&amp; L (HRP) (ab6721, Abcam, Cambridge, UK, dilution 1:1000). Sections were washed five times in TBS buffer. The reaction was visualized for 3 min using DAB Quanto (Epredia™ DAB Quanto Detection System, Montréal, QC, Canada). Contrast staining was performed using Mayers hematoxylin (Sigma-Aldrich, St. Louis, MO, USA). Immunoreaction was verified with negative controls subjected to identical immunohistochemical staining, excluding the use of primary antibodies. Eight areas of interest (ROI) within the villi for each animal were selected for ZIP1 and ZIP4 analysis. The ROIs had the same dimensions in each analyzed image, within each animal and each group. IHC profiler (Image J plugin) was used for quantitative IHC analysis using color deconvolution and computerized pixel profiling, which led to the automatic scoring of the corresponding image. The IHC Profiler generates a DAB image histogram profile corresponding to the number of pixel intensity counts and automatically calculates the score. The result is displayed in a semi-quantitative manner and is used to calculate the optical density of the IHC.

### 2.6. ZnT1 and Metallothionein Liver Concentration (n = 8)

The assessment of MT-1, MT-2, MT-3, and ZnT1 concentration in the liver (right median lobe) was performed using an enzyme-linked immunoassay (ELISA) kit from Enlibio Biotech Co., Ltd. (Wuhan, China) based on a double-antibody sandwich technique. MT-1, MT-2, and MT-3 levels were measured following the manufacturer’s protocol. The 96-well microtiter plates were read at 450 and 570 nm using a spectrophotometer (Synergy HTX multimode reader machine; BioTek Instruments Inc., Winooski, VT, USA).

### 2.7. TBARS, SOD, and GSH-Px Liver Concentration (n = 8)

TBARS, SOD, and GSH-Px levels were determined using an enzyme-linked immunoassay (ELISA) kit from BT-lab (Shanghai, China) following the kit’s instructions. The 96-well microtiter plates were read at 450 and 570 nm using a spectrophotometer (Synergy HTX multimode reader machine; BioTek Instruments Inc., Winooski, VT, USA).

### 2.8. Zinc, Copper, and Iron Liver Concentration (n = 8)

Tissues (±0.5 g each) were weighted with an accuracy of 0.0001 g and mineralized in the Magnum II microwave apparatus (ERTEC, Kołbaskowo, Poland) using 2 mL of 30% H_2_O_2_ Suprapure^®^ solution and 4 mL of concentrated 65% HNO_3_ Suprapure^®^ solution (both Merck, Darmstadt, Germany) with three stages (with successively increasing parameters: temperature 250–300 °C, pressure 20–35 bar, time 10–20 min, and microwave power of 60–100%). After this procedure, solutions were evaporated on a heating plate at 150 °C, and an almost dry residue was filled up to 25 mL with four-times-distilled water. The trace elements Cu, Fe, and Zn were measured using flame atomic absorption spectroscopy (F-AAS) with the AAS iCE3300 Thermo Scientific™ spectrophotometer (Oxford, UK).

### 2.9. Statistical Analysis

All data are presented as mean and standard deviation (SD) and were analyzed by one-way ANOVA followed by Tukey’s post hoc test for significant differences to correct multiple comparisons, using Statistica^®^ ver. 13.3 software packages (StatSoft Inc., Tulsa, OK, USA). A significance level (*p*-value) of less than 0.05 was considered statistically significant. Each mouse was an experimental unit (*n* = 8 per group). Normal distribution of the data was confirmed using the Shapiro–Wilk W test, and equality of variance was verified using Levene’s test. Similarly, the normality of distribution and homogeneity of variance was checked for measurements of the percentage of collagen tissue in the liver. In this case, the Kruskal–Wallis test was applied using Statistica^®^ ver. 13.3 software packages (StatSoft Inc., Tulsa, OK, USA). The results were visualized with graphs using GraphPad (Prism, Boston, MA, USA).

The model employed in the analysis was as follows:

$$Y_{ij}=\mu +\alpha_{i}+\delta_{ij}$$

where *Y_ij_* represents the observed dependent variable, μ is the overall mean, α*_i_* is the group effect, and δ*_ij_* is the random error.

## 3. Results

### 3.1. Mice Body, Thymus, and Spleen Weight

**Figure 2:**
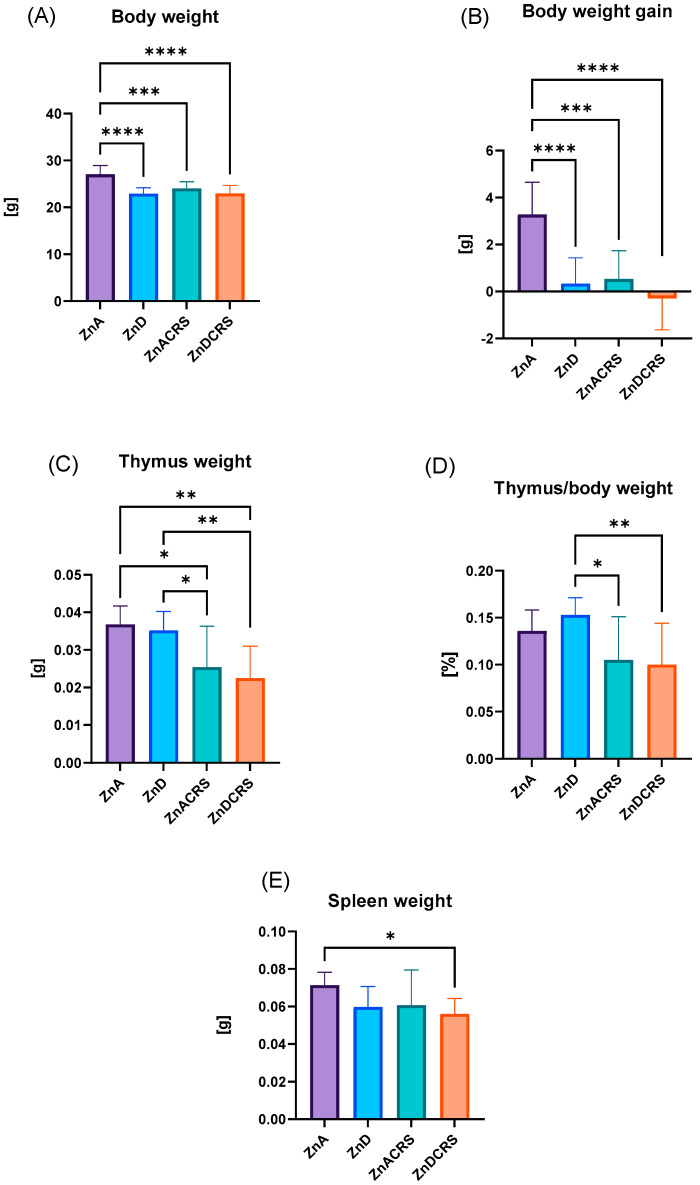
The effect of the ZnD, CRS, and ZnDCRS on mice body weight (**A**) and body weight gain (**B**); thymus and (**C**) thymus/body weight (**D**), and spleen weight (**E**). * *p* < 0.05, ** *p* < 0.01, *** *p* < 0.001, and **** *p <* 0.0001. Columns represent means, and whiskers represent standard deviations. *N* = 8. ZnA—diet containing 50 mg Zn/kg Zn; ZnD—diet containing < 3 mg Zn/kg Zn for 4 weeks; CRS—3 weeks of chronic stress (3 h/d).

After 3 weeks of a zinc-restricted diet and 3 weeks of a restraint stress protocol, we observed significant differences in animals’ body weight and body weight gain (Figure 2A,B). Mice from the ZnA group weighed an average of 27.05 ± 1.85 g, while ZnD was 22.93 ± 1.26 g, ZnACRS was 24.01 ± 1.43 g, and ZnDCRS was 22.95 ± 1.73 g. This shows that restraint stress, zinc restriction, and both factors simultaneously strongly lower mice’s body weight.

During the 21 days of the experiment, the body weight gains of the control mice increased (3.28 ± 1.37 g), while the body weight gains of the ZnD (0.34 ± 1.09 g), ZnACRS (0.53 ± 1.21 g), and ZnDCRS (−0.29 ± 1.34 g) mice dropped significantly. The animals’ average body weight at the experiment’s beginning did not differ significantly between groups.

Zinc restriction did not affect thymus weight (0.035 ± 0.005 g). However, the results show significantly reduced thymus weight in mice exposed to CRS (0.025 ± 0.011 g) and CRS combined with zinc restriction (0.022 ± 0.008 g) in comparison to the ZnA (0.037 ± 0.005 g) and ZnD groups (Figure 2C). We also evaluated the thymus ratio, which is the thymus weight/body weight ratio. It was the highest in the ZnD group (0.15 ± 0.01%), which significantly differed from ZnACRS (0.10 ± 0.05%) and ZnDCRS (0.10 ± 0.04%) (Figure 2D). The spleen of mice in the ZnDCRS (0.056 ± 0.001 g) group was reduced in size in comparison with the ZnA group (0.071 ± 0.007 g) (Figure 2E). The animal treatments did not affect the average % of the spleen/body weight ratio between ZnA (0.26 ± 0.01%), ZnD (0.26 ± 0.06%), ZnACRS (0.25 ± 0.07%), or ZnDCRS (0.25 ± 0.04%) groups.

### 3.2. Hematoxylin and Eosin Staining of the Thymus and Spleen

**Figure 3:**
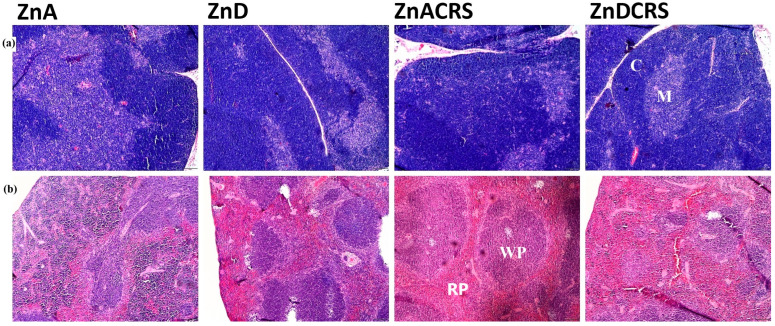
Hematoxylin–eosin staining of the thymus (**a**) and spleen (**b**). Magnification: 5×. ZnA—diet containing 50 mg Zn/kg; ZnD—diet containing < 3 mg Zn/kg for 4 weeks; CRS—3 weeks of chronic stress (3 h/d). Abbreviations: thymus, ZnDCRS (M—medulla; C—cortex); spleen, ZnACRS (WP—white pulp; RP—red pulp).

Hematoxylin and eosin staining was performed to assess potential histopathological changes in the thymus and spleen. The microscopic analysis revealed no significant abnormalities in the structure of either organ (Figure 3a,b). No inflammation or necrosis changes were observed in either tissue.

### 3.3. Morphometric Measurements of the Intestines

**Figure 4:**
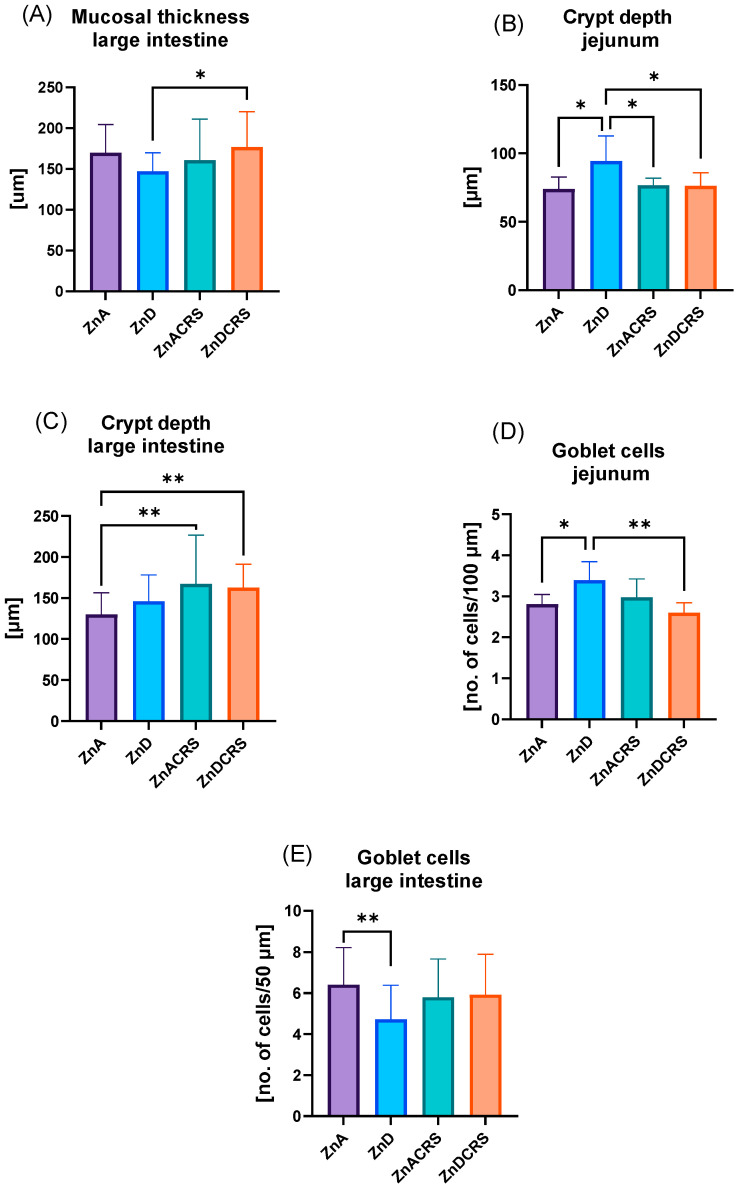
The effect of the ZnD, CRS, and ZnDCRS on mucosal thickness (**A**) and crypt depth (**C**) in the large intestine, crypt depth in the jejunum (**B**), number of goblet cells in the jejunum (**D**), and large intestine (**E**). * *p* < 0.05; ** *p* < 0.01. Columns represent means, and whiskers represent standard deviations. ZnA—diet containing 50 mg Zn/kg; ZnD—diet containing < 3 mg Zn/kg for 4 weeks; CRS—3 weeks of chronic stress (3 h/d).

As shown in Figure 4A–D, stress and zinc restriction affected morphometric changes in the intestines. The colonic mucus layer was significantly thicker in ZnDCRS mice (176.5 ± 7.96 μm) compared to the ZnD group (146.9 ± 4.21 μm). Hematoxylin–eosin with Alcian blue staining demonstrated an increase in the crypt depth in the ZnD group (94.3 ± 6.9 μm) vs. ZnA (73.97 ± 3.3 μm), ZnACRS (76.51 ± 2 μm), and ZnDCRS (76.15 ± 3.7 μm) in the jejunum and ZnACRS (167.3 ± 10.82 μm) and ZnDCRS (162.6 ± 5.22 μm) vs. ZnA (130 ± 4.9 μm) in the large intestine.

The number of goblet cells/100 μm in the jejunum was increased in the ZnD (3.39 ± 0.17) group compared to ZnA (2.81 ± 0.08) and ZnDCRS (2.59 ± 0.09) mice. In the large intestine, the opposite results were observed. In response to zinc restriction, goblet cell count/100 μm decreased (4.72 ± 0.34) compared to the ZnA group (6.11 ± 0.35).

Treatment with zinc restriction, CRS, and combined factors did not affect the mucosal thickness, crypt depth, crypt width, villi/crypt ratio, and number of goblet cells in the particular part of the gastrointestinal tract (Table S1—Supplementary Materials).

**Figure 5:**
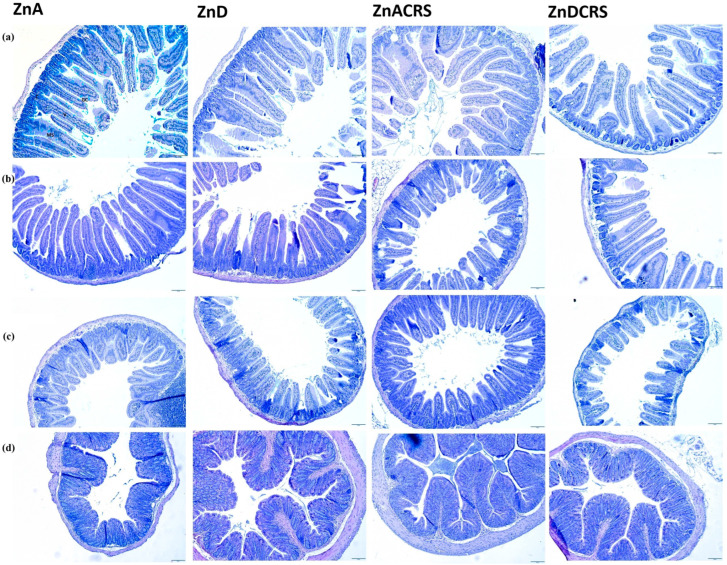
Small and large intestines histology (hematoxylin–eosin with Alcian blue staining): (**a**) duodenum, (**b**) jejunum, (**c**) ileum, and (**d**) colon in the ZnA, ZnD, ZnACRS, and ZnDCRS groups. Magnification 2.5×. ZnA—diet containing 50 mg Zn/kg; ZnD—diet containing < 3 mg Zn/kg for 4 weeks; CRS—3 weeks of chronic stress (3 h/d). Abbreviations: duodenum, ZnACRS (V—villi; C—crypt; GC—goblet cells; MS—mucosa).

Figure 5 shows hematoxylin–eosin staining with Alcian blue of the duodenum, jejunum, ileum, and colon in the ZnA, ZnD, ZnACRS, and ZnDCRS groups.

### 3.4. ZIP1 and ZIP4 Expression in the Jejunum

**Figure 6:**
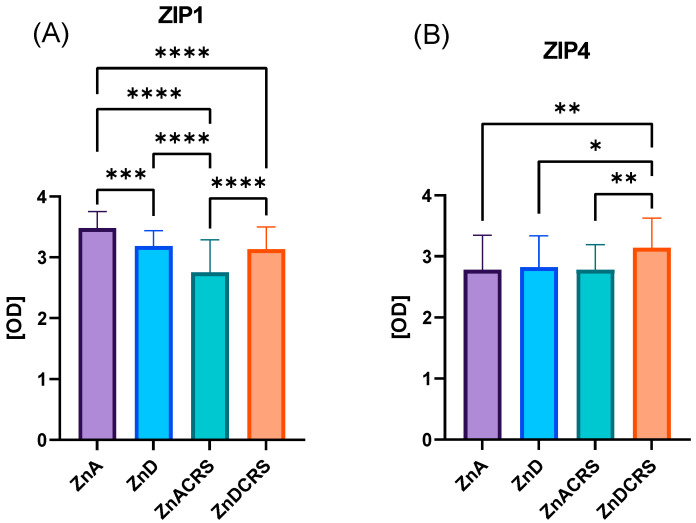
The effect of the ZnD, CRS, and ZnDCRS on the optical density of zinc transporters ZIP1 (**A**) and ZIP4 (**B**) in the jejunum * *p* < 0.05, ** *p* < 0.01, *** *p* < 0.001, and **** *p <* 0.0001. Columns represent means, and whiskers represent standard deviations. ZnA—diet containing 50 mg Zn/kg; ZnD—diet containing < 3 mg Zn/kg for 4 weeks; CRS—3 weeks of chronic stress (3 h/d).

It was found that ZIP4 protein expression was the highest in the jejunum of the ZnDCRS (3.143 ± 0.07 OD) mice compared to ZnA (2.782 ± 0.08 OD), ZnD (2.882 ± 0.07 OD) and ZnACRS (2.781 ± 0.05 OD) groups. Both were subjected to restraint stress and reduced zinc content in the diet, and both these factors together led to reduced ZIP1 expression: ZnA (3.482 ± 0.04 OD), ZnD (3.184 ± 0.04 OD), ZnACRS (2.754 ± 0.08 OD), and ZnDCRS (3.135 ± 0.05 OD) (Figure 6).

### 3.5. The Percentage of Collagen Fibers in the Liver

**Figure 7:**
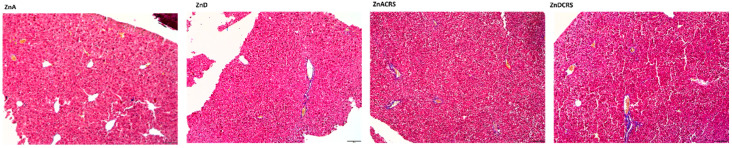
Liver Masson-Goldner trichrome staining with Aniline blue in the ZnA, ZnD, ZnACRS, and ZnDCRS groups. Magnification 5×. ZnA—diet containing 50 mg Zn/kg; ZnD—diet containing < 3 mg Zn/kg for 4 weeks; CRS—3 weeks of chronic stress (3 h/d).

As observed by Masson-Goldner trichrome staining, there were more collagen fibers (*p* = 0.0041) in the ZnD group (1.824 ± 0.47%) vs. ZnA (0.658 ± 0.46%) and ZnACRS (0.694 ± 1.70%) groups. Group ZnDCRS (1.166 ± 0.95%) did not differ from other treatments. The image of the Masson-Goldner trichrome staining with Aniline is shown in Figure 7.

### 3.6. The Content of Metallothioneins and ZnT1 Transporter in the Liver

**Figure 8:**
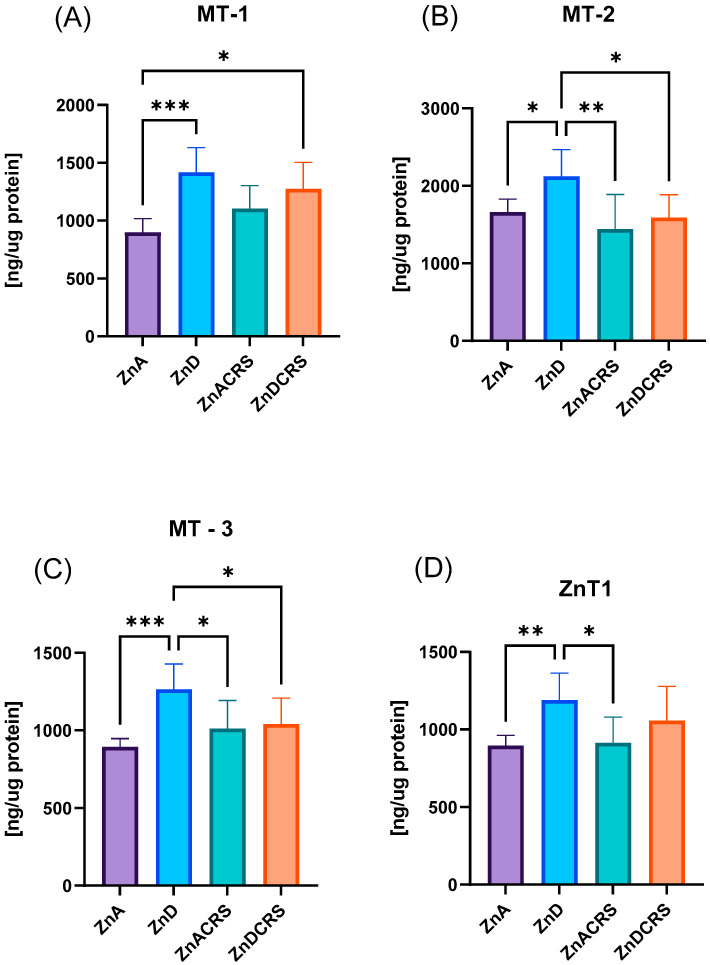
The effect of the ZnD, CRS, and ZnDCRS on the concentrations of MT-1 (**A**), MT-2 (**B**), MT-3 (**C**), and ZnT1 (**D**) transporter in the liver. * *p* < 0.05, ** *p* < 0.01, and *** *p* < 0.001. Columns represent means, and whiskers represent standard deviations. ZnA—diet containing 50 mg/kg Zn; ZnD—diet containing < 3 mg/kg Zn for 4 weeks; CRS—3 weeks of chronic stress (3 h/d).

The concentrations of MT-2 and MT-3 (Figure 8B,C) were the highest in the ZnD group (MT-2: 2124 ± 122 ng/µg protein, MT-3: 1265 ± 57.59 ng/µg protein) compared to the ZnA (MT-2: 1663 ± 58.90 ng/µg protein, MT-3: 896 ± 18.50 ng/µg protein), ZnACRS (MT-2: 1443 ± 157.8 ng/µg protein, MT-3: 1012 ± 64.39 ng/µg protein), and ZnDCRS (MT-2: 1591 ± 104.5 ng/µg protein, MT-3: 1041 ± 59.45 ng/µg protein) groups. Zinc restriction in the diet alone and in combination with stress significantly increased MT-1 in both ZnD (1418 ± 75.34 ng/µg protein) and ZnDCRS groups (1275 ± 80.89 ng/µg protein) vs. control group (899 ± 42.15 ng/µg protein) (Figure 8A).

ZnA (898.6 ± 22.38 ng/µg protein) and ZnACRS (915.0 ± 57.93 ng/µg protein) groups had lower liver ZnT1 protein expression than the ZnD group (1190 ± 61.27 ng/µg protein) (Figure 8D).

### 3.7. TBARS, SOD, and GSH-Px Liver Concentration

**Figure 9:**
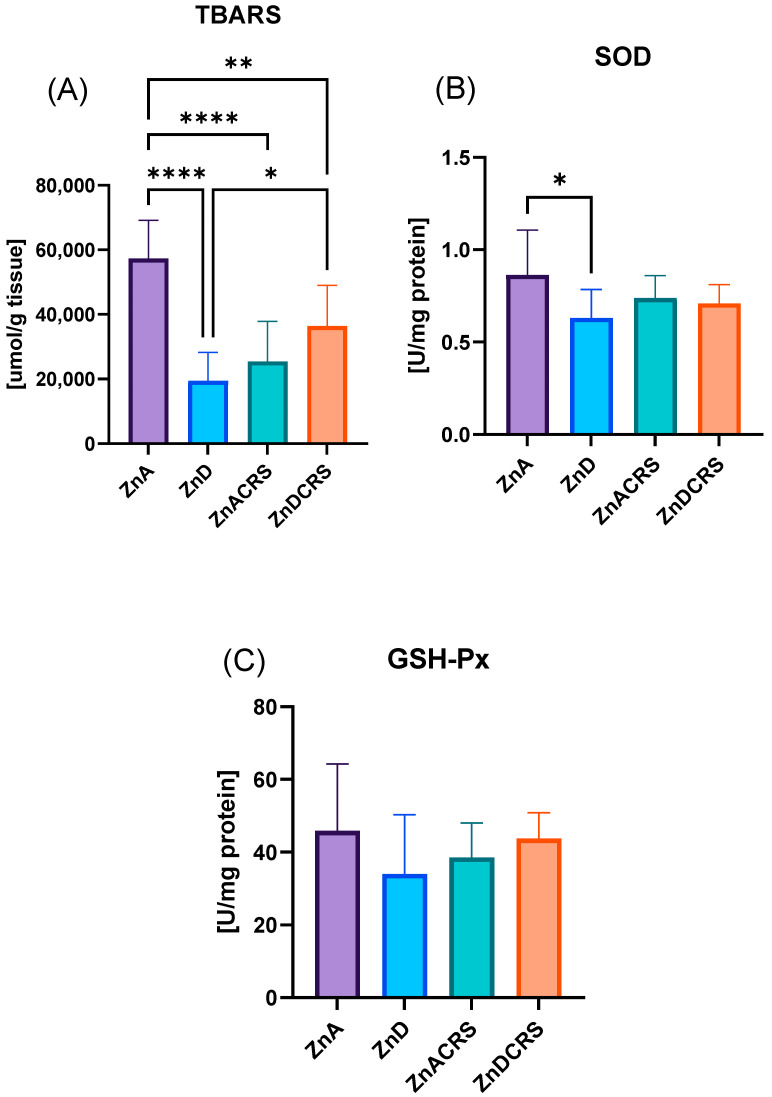
The effect of the ZnD, CRS, and ZnDCRS on the concentrations of TBARS (**A**), SOD (**B**), and GSH-Px (**C**) in the liver. * *p* < 0.05, ** *p* < 0.01, and **** *p* < 0.0001. Columns represent means, and whiskers represent SD. ZnA—diet containing 50 mg Zn/kg; ZnD—diet containing < 3 mg Zn/kg for 4 weeks; CRS—3 weeks of chronic stress (3 h/d).

The oxidative stress (Figure 9A,B) measured indirectly by TBARS level was the highest in the ZnA (57,281 ± 11,913 umol/g tissue) group compared to the rest of the treatments (ZnD 19,417 ± 8813; ZnACRS 25,433 ± 12,372; ZnDCRS ± 36,350 umol/g tissue). A significantly reduced TBARS level was seen in the ZnD mice compared to ZnDCRS. The highest activity of SOD was observed in the ZnA (0.862 ± 0.24 U/mg protein) vs. ZnD (0.613 ± 0.15 U/mg protein) group. No significant differences were found in GSH-Px activity (ZnA 42.95 ± 18.32 U/mg protein, ZnD 34.06 ± 16.22 U/mg protein, ZnACRS 38.54 ± 9.51 U/mg protein, ZnDCRS 43.75 ± 7.06 U/mg protein).

### 3.8. Zinc, Copper, and Iron Liver Concentration

**Figure 10:**
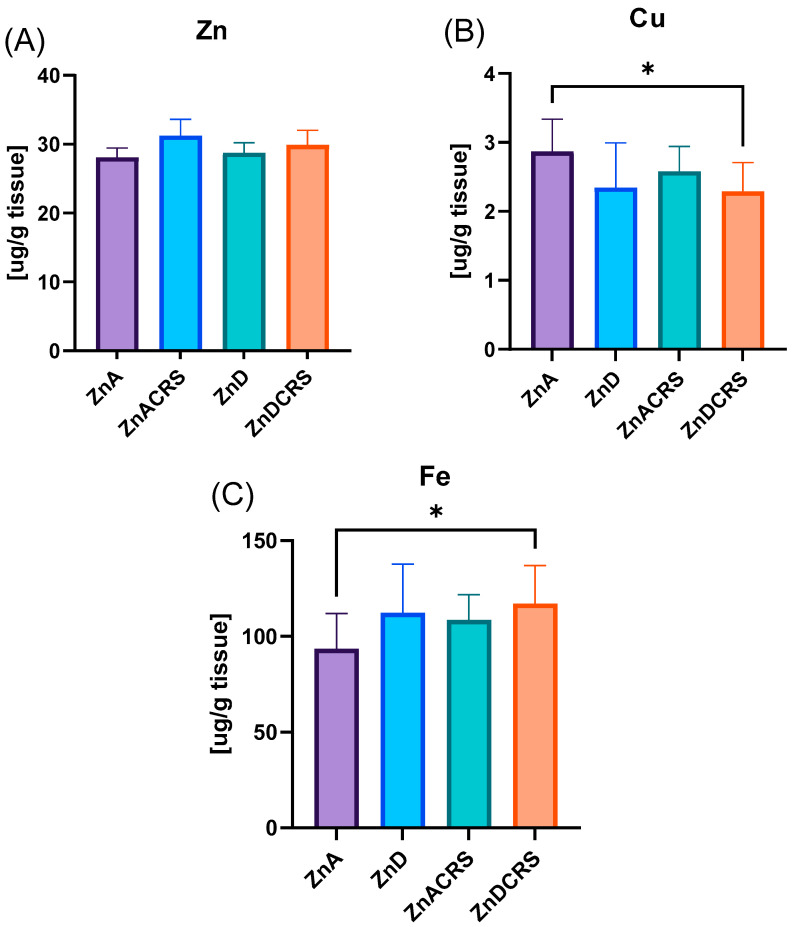
The effect of the ZnD, CRS, and ZnDCRS on the concentration of Zn (**A**), Cu (**B**), and Fe (**C**) in the liver. * *p* < 0.05. Columns represent means, and whiskers represent standard deviations. ZnA—diet containing 50 mg Zn/kg; ZnD—diet containing < 3 mg Zn/kg for 4 weeks; CRS—3 weeks of chronic stress (3 h/d).

Animals in the group receiving a diet with reduced zinc content had lower copper concentrations in the liver compared to the ZnA group (ZnA 2.86 ± 0.47 µg/g tissue; ZnDCRS 2.29 ± 0.42 µg/g tissue) and higher iron concentrations (ZnA 93.6 ± 18.36 µg/g tissue; ZnDCRS 117 ± 19.94 µg/g tissue). No significant differences were found in zinc concentration (ZnA 28.09 ± 6.04 ug/g tissue, ZnACRS 31.26 ± 7.42 ug/g tissue, ZnD 28.74 ± 9.51 ug/g tissue, ZnDCRS 29.89 ± 6.04 ug/g tissue) (Figure 10).

## 4. Discussion

Our study demonstrated that both CRS and zinc restriction, individually and in combination (ZnDCRS), significantly impacted the experimental animals’ body weight and weight gain. Similar findings have been reported in other studies on rodents, showing reduced body weight and weight gain under stress conditions [25,34,35,36,37,38,39,40,41]. These effects may be attributed to a reduced feed intake at the beginning of the experiment, followed by increased energy expenditure as the animals attempt to maintain proper body temperature during the stress procedure. Interestingly, the body weight did not return to the weight of control animals for an extended time [42]. The second factor, zinc restriction, can cause anorexic-like behavior and disrupt appetite regulation. Zinc is crucial in the synthesizing neurotransmitters and controlling appetite and taste perception. The literature data indicate that its deficiency impairs communication between neurons involved in appetite regulation, leading to reduced food intake [43]. These findings highlight the importance of zinc in maintaining both neural and metabolic health and physiological resilience under stress.

Psychological factors such as stress undoubtedly affect the immune system and immune organ functioning. Prolonged stress can weaken the immune system, mainly due to the release of stress hormones such as cortisol [44]. In our studies, CRS induces thymus atrophy. ZnDCRS, in addition to inducing thymic atrophy, also reduces spleen weight. In 1998, Shi et al. [45] showed that limiting zinc intake decreased the relative spleen size to body weight and the overall number of spleen cells. Using social defeat stress, some authors observed an overall increase in spleen weight in stressed mice, which was explained by increased mobilization of leukocytes and accumulation of bone marrow-derived hematopoietic progenitors [46,47,48]. The thymus/body ratio was the highest in the ZnD group, as those mice lost weight but did not suffer from thymus atrophy, contrary to the ZnACRS and ZnDCRS mice. It is known that psychological stress causes thymic involution due to increased glucocorticoid (GC) secretion from adrenal glands [16,17]. The thymus is also one of the several extra-adrenal organs that produce de novo and metabolizing glucocorticoids locally (called immunosteroids). However, their role is still not fully understood [49]. They probably contribute to thymocyte apoptosis. A high level of ACTH (corticotropin-releasing hormone) stimulates GC synthesis in the adrenals but has the opposite effect on the GC produced in the thymus [38]. The other theories of thymus atrophy indicate the involvement of other factors, such as the synthesis of IL-6 and TNF-α and leptin deficiency [21,50]. Other researchers have confirmed the thymus’s sensitivity to stress [50,51]. The advantages of this process are still unknown. However, it can be speculated that it is an adaptive response to prevent autoimmune reactions due to catabolism and the release of self-antigens [52]. Zinc status is essential for proper thymus functioning, as it regulates the activity of the immunoregulatory peptide thymulin. Its lower production led to immunosuppression, as it disrupts the maturation of lymphocytes T [53]. Other researchers have noted that zinc deficiency reduces T-cell counts, attributed to thymic atrophy. Thymus atrophy is linked not only to reduced thymulin activity but also to lower levels of IL-2 and alterations in IL-2-induced signaling pathways, all of which contribute to increased lymphocyte apoptosis and reduced proliferation [54,55,56,57].

Our findings, along with those of previous studies, underscore the essential role of zinc in immune regulation and suggest that combined stress and ZnD impair thymic and splenic function, leading to a more substantial reduction in immunity than either factor alone.

In response to zinc deficiency, the liver and intestines may activate adaptive mechanisms to enhance zinc uptake and utilization. Zinc is mainly absorbed in the duodenum and proximal part of the jejunum and is transported to enterocytes by specific transporters expressed in the apical membrane [58]. Its regulation occurs at several levels: intestinal absorption, gastrointestinal excretion, urinary excretion, and cellular retention. A deficit in dietary zinc intake is compensated by reducing fecal Zn excretion, increasing absorption efficiency to almost 100% [59,60,61]. Our results showed that zinc restriction reduced mucosal thickness in the large intestine compared to the ZnDCRS. The CRS group of animals had increased crypt depth, while the ZnD group had fewer goblet cells in the large intestine. The jejunum was also affected by low zinc, with the highest goblet cell count and crypt depth observed in this region. Numerous studies have reported that dietary zinc improves and also prevents the reduction in intestinal integrity during chronic inflammatory bowel disease, infectious diarrhea [62,63], and heat stress [64] and also reduces intestinal permeability [65]. Impaired intestinal barrier integrity provides a gateway for pathogenic bacteria and other antigenic substances in the intestine to direct contact with intestinal epithelial cells, stimulating ongoing submucosal inflammation [66]. Studies have reported that zinc taken in with food acts mainly at the level of tight junctions. In vitro studies have shown that Caco-2 cells cultured in a zinc-depleted medium exhibited reduced tight junctions and an impaired cytoskeleton [67]. A zinc-deficient diet affects intestinal histology by increasing *MUC2* gen activity and producing mucins consisting mainly of short O-glycans, which disturbs their stability [68]. Stress can also alter mucus secretion. One study evaluated the effect of immobilization stress on rat colonic mucus release and mast cell degranulation. Immobilization caused a significant increase in mucin release from colonic mucosal explants as well as a decrease in the number of goblet cells at the histological level, accompanied by an increase in rat colonic mucosal mast cell protease II, PGE 2,cu, and cyclooxygenase-2 mRNA levels [69,70]. Zinc is also essential in the formation of the intestinal mucosa. Livesey et al. [71] showed that Zn deficiency in rats was associated with a decreased crypt/villi ratio and a lower rate of division of crypt cells in the jejunum and mucosal cells in the jejunum. This resulted in a significant decrease in the net influx of new cells into the villi. It is thought that the reduced influx of cells into the villi may be responsible for the morphological and functional changes observed in the small intestine of rats fed a low-Zn diet. Another factor analyzed was CRS. One study indicated that stress has both central and peripheral effects, thus promoting both mental illnesses and peripheral intestinal diseases [72]. Stress in humans is associated with exacerbation of intestinal diseases, including irritable bowel syndrome or inflammatory bowel disease. Stress has been proven to induce colitis based on studies in rat and mouse models [73,74]. ACTH, released after acute stress in rats, also induces intestinal epithelial pathophysiology [13]. A study by Saunders et al. [75] found that acute stressors profoundly affect intestinal epithelial physiology, stimulating ion secretion and reducing barrier function (increasing permeability). The study of Santos et al. [76] concluded that chronic exposure to stress causes epithelial barrier dysfunction, inflammation, and metabolic abnormalities in rats and mice, with the observed changes involving both the small and large intestines. It was shown that chronic stress caused epithelial barrier defects and epithelial mitochondrial damage, with concomitant proliferation and activation of mucosal mast cells. The above studies showed that chronic psychological stress causes changes in the jejunum, ileum, and colon morphology; increased permeability; increased ion secretion; binding of luminal bacteria to the surface epithelium; and changes in the intestinal mucosa. Exposure to chronic stress impaired the morphology of villi and crypts in the ileum and jejunum of rats. It impaired the function of the small intestinal barrier in the study conducted by Khan et al. [77]. Interestingly, zinc supplementation in this model prevented or alleviated these stress-induced changes. In the zinc-supplemented groups, there was a noticeable increase in villi length in the jejunum and ileum. There was also a noticeable effect of zinc on an increase in the number of goblet cells and a decrease in their number upon exposure to stress. Our results indicate that both ZnD and CRS independently impair intestinal integrity, as seen in reduced mucosal thickness and altered goblet cell distribution, while the combination of ZnD and CRS (ZnDCRS) leads to even more pronounced disruptions. These results align with previous studies highlighting zinc’s essential role in maintaining gut morphology and barrier function under stress, suggesting that ZnDCRS conditions may activate distinct adaptive responses in zinc uptake mechanisms that partially compensate for these stressors but fail to fully restore intestinal health.

The immunohistochemistry of the jejunum showed upregulation of ZIP4 in the ZnDCRS group, while ZIP1 was downregulated due to CRS and reduced zinc levels. This indicates that stress signaling may influence molecules that enhance ZIP4 expression, aiding cellular adaptation. ZIP4, located in the apical membrane of intestinal villi, is crucial for dietary zinc uptake and is regulated by zinc availability. During zinc deficiency, ZIP4 expression increases to raise zinc absorption [78,79]. ZIP4’s importance is highlighted by its role in acrodermatitis enteropathica [80].

The liver is one of the major zinc storage sites, and as mentioned earlier, it plays a crucial role in regulating zinc homeostasis. Besides the ZIP and ZnT family proteins, MT is also essential for maintaining intracellular zinc homeostasis, as mentioned earlier. Our studies showed that ZnD alone and in combination with CRS significantly increased MT-1 synthesis. Meanwhile, MT-2,3 synthesis in the liver was significantly stimulated only in response to ZnD. In our study, CRS appears to act as an inhibitor of the synthesis of metallothionein 2 and 3 during 28 days of zinc restriction (ZnDCRS group). Based on the outcomes of the different studies, 16 days of zinc deficiency significantly lowered MT concentration in the rat liver. However, it did not alter hepatic Zn concentrations [81]. These findings indicated that Zn concentration is not a determinant of MT concentration in the rat liver. Our results are in agreement with these studies. Although the zinc level was not changed, the MT level differed significantly. MT may influence the chemical form in which Zn is available within the cell and the scope of its action. Waalkes [82] investigated the effect of continuous dietary zinc deficiency (9 weeks) on the hepatic zinc concentration, which was not affected by the diet. The MT level was cadmium dose-dependent, regardless of zinc status. Cui et al. [83] showed that Zn-deficient rats had significantly reduced hepatic MT and zinc concentrations in plasma and liver. Although zinc deficiency typically lowers MT levels, our study showed the opposite effect. This may result from compensatory mechanisms during chronic restriction (28 days), where increased metallothionein gene transcription helps enhance zinc uptake or redistribution within cells to prevent further depletion. Additionally, chronic inflammatory conditions such as zinc restriction can further stimulate metallothionein synthesis [84]. Higher MT-1, MT-2, and MT-3 concentrations suggest a compensatory response, but it is difficult to determine whether it is a long-term or short-term phenomenon. One of the authors also explored the topic of the susceptibility of different stresses in mice to produce MT in response to Zn. The C57BL/6 and C57BL/10 strains were identified as accumulating the lowest levels of MT following Zn administration. Interestingly, MT binding to different metals has different times of degradation. Cd-bound MT has a long half-life, between 2.8 and 5.8 days, while Cu-MT has a half-life of 12–17 h [85].

Zinc transporters are essential for maintaining zinc balance in the intestines and other organs, such as the liver. The ZnT1 transporter increases its expression in response to zinc restriction but not under stress, highlighting its role in intracellular zinc homeostasis inhibited by stress. Elevated ZnT1 levels correspond with metallothionein expression, suggesting it helps maintain the liver’s zinc balance during zinc deficiency. However, this mechanism does not operate under stress. Another study also found increased ZnT1 expression in the livers of animals on a low-zinc diet (7 mg Zn/kg), while a contrasting decrease was noted in animals receiving less than 1 mg Zn/kg [86].

Control animals showed enhanced liver susceptibility to oxidative stress measured by TBARS level. Zinc is essential for enzymes like superoxide dismutase (SOD), which was the highest in the control group due to adequate zinc intake. Zinc deficiency may impair antioxidant defenses, affecting oxidative stress responses. However, lower TBARS levels suggest other mechanisms are engaged, possibly linked to significant metallothionein expression in zinc-restricted animals, indicating its potential protective role against oxidative stress and lipid peroxidation. This relationship warrants further investigation. Reduced feed intake or increased caloric demand from stress may alter metabolic priorities and liver lipid composition, affecting membrane susceptibility to peroxidation. Calorie restriction significantly decreases the markers of protein oxidation, glycoxidation, and lipoxidation and affects the fatty acid composition of liver mitochondria, according to Gomez et al. [87]. The authors noted a moderate decrease in membrane fatty acid unsaturation in calorie-restriction groups, contributing to lower oxidative stress and showing a strong relationship between lipid profile and the sensitivity of membranes to lipid peroxidation, similar to other research [88,89].

Our results indicate that zinc restriction alters the concentrations of trace elements like copper and iron in the mouse liver despite unchanged zinc levels. Yu et al. [90] revealed the effect of zinc deficiency on the disturbance of the homeostasis of 12 minerals and trace elements in rats’ serum, liver, feces, and urine. A low-zinc diet (10 mg/kg) decreased the concentrations of Zn, Mg, Cu, Se, and K in the liver, while Fe remained unchanged. The observed decreased urinary Cu suggests its higher renal reabsorption due to zinc deficiency, although fecal excretion of Cu and Fe may be increased. Considering our own experience and that of other authors, it can be assumed that at the expense of copper absorption, zinc absorption is thought to increase during zinc deficiency. The discovery that zinc atoms may compete with copper for their absorption in the gastrointestinal tract led to the introduction of zinc in the therapy of Wilson’s disease. Although high doses of zinc are used in this therapy, it was also described that zinc-deficient humans absorb zinc more efficiently, whereas humans with a high-zinc diet have reduced zinc absorption; therefore, it cannot be ruled out that a prolonged low-zinc diet blocks copper absorption [91]. Moreover, increased MT-1 levels observed in both the ZnD and ZnDCRS groups in the liver may be responsible for maintaining zinc homeostasis during dietary zinc restriction. Lower copper levels in the liver could lead to higher iron levels due to impaired activity of the copper-dependent enzyme ceruloplasmin that is responsible for converting Fe^2+^ to Fe^3+^, which is important for unbalanced iron export and metabolism.

Our work is a broad approach to the reflection of the problem of zinc restriction and chronic stress throughout the body. It opens the possibility for further detailed exploration of these problems, which are increasingly relevant in the modern world. Both zinc restriction and stress significantly affected body weight, immune organ weight, zinc and metallothionein homeostasis, and oxidative stress response in experimental animals. Stress and zinc restriction leads to lower body weight and altered body weight gain. Moreover, this relationship was not obvious at all. Contrary to our expectations, zinc restriction in C57BL/6J mice did not lead to a sharp decrease in zinc or metallothionein levels in the liver. Our study revealed compensatory mechanisms that help maintain zinc concentrations within normal ranges, influencing its bioavailability for cellular biochemical processes.

## 5. Conclusions

In this study, we aimed to investigate the systemic effects of combined zinc restriction and chronic stress, focusing on changes beyond the brain. Our findings provide a comprehensive view of how this model impacts peripheral organs (intestines, thymus, spleen, and liver), highlighting the importance of considering both nutritional and stress-related factors in the context of overall physiological health.

## Data Availability

The raw data supporting the conclusions of this article will be made available by the authors on request.

## Supplementary Materials

The following supporting information can be downloaded at https://www.mdpi.com/article/10.3390/nu16223934/s1. Table S1: Effects of zinc deficiency and chronic restraint stress on intestinal histomorphometry in the duodenum, jejunum, and ileum.
