# Supplementary material for: Dietary Zinc Restriction and Chronic Restraint Stress Affect Mice Physiology, Immune Organ Morphology, and Liver Function
Source: Nutrients. 2024 Nov 18;16(22):3934. doi: 10.3390/nu16223934 (PMC11597199; doi:10.3390/nu16223934)
Supplement: Supplementary file 1 [file nutrients-16-03934-s001.zip › nutrients-3207059-supplementary.pdf]

Table S1.

Item	ZnA	ZnD	ZnACRS	ZnDCRS	P
**Duodenum**					
Mucosal thickness. µm<br>SD	492±<br>47.77	589.1±<br>92.13	586.3±<br>42.73	564.9±<br>78.33	0.1
Villi width. µm<br>SD	65.85±<br>9.91	67.33±<br>3.83	77.83±<br>14.78	68.32±<br>2.95	0.12
Crypt depth. µm<br>SD	76.78±<br>11.72	82.77±<br>13.53	84.58±<br>13.93	87.44±<br>14.77	0.58
Crypt width. µm<br>SD	28.51±<br>3.29	30.73±<br>4.68	28.45±<br>3.01	30.65±<br>5.47	0.63
Villi/crypt ratio<br>SD	6.08±<br>0.36	5.95±<br>0.92	6.04±<br>1.10	5.77±<br>0.80	0.94
No. of goblet cells/100 µm section<br>SD	3.21±<br>0.28	3.09±<br>0.41	3.10±<br>0.35	3.34±<br>0.41	0.61
**Jejunum**					
Mucosal thickness. µm<br>SD	423.3±<br>49.4	449±<br>69.86	404.8±<br>61.87	397.8±<br>72.7	0.46
Villi height. µm<br>SD	356.5±<br>53.97	356.8±<br>53.17	338.5±<br>53.67	338.5±<br>60.68	0.86
Villi width. µm<br>SD	58.31±<br>7.5	58.6±<br>6.3	54.99±<br>6.56	53.27±<br>5.16	0.35
Crypth width. µm<br>SD	25.33±<br>3.67	28.52±<br>3.97	24.7±<br>4.04	25.26±<br>2.71	0.22
Villi/crypt ratio<br>SD	4.85±<br>0.74	3.91±<br>0.89	4.47±<br>0.89	4.45±<br>0.6	0.19
**Ileum**					
Mucosal thickness. µm<br>SD	275.4±<br>11.51	257±<br>57	261.4±<br>39.53	249.8±<br>64.27	0.77
Villi height. µm<br>SD	206.2±<br>14.72	185.4±<br>36.65	201±<br>24.3	185.8±<br>51.22	0.62
Villi width. µm<br>SD	56.44±<br>5.36	59.89±<br>7.34	56.53±<br>5.42	49.59±<br>6.26	0.06
Crypt depth. µm<br>SD	76.37±<br>11	82.21±<br>19.61	77.19±<br>17.09	68.55±<br>11.1	0.58
Crypth width. µm<br>SD	22.99±<br>3.78	26.73±<br>5.50	26.16±<br>2.92	26.17<br>2.32	0.28
Villi/crypt ratio<br>SD	2.74±<br>0.37	2.28±<br>0.21	2.66±<br>0.36	2.92<br>0.27	0.06
No. of goblet cells/100 µm section<br>SD	3.75±<br>0.46	3.71±<br>0.92	3.62±<br>0.48	3.43<br>0.32	0.78
